# A convenient, reliable, and fast acoustic pressure field measurement method for magnetic resonance-guided high-intensity focused ultrasound systems with phased array transducers

**DOI:** 10.1186/s40349-018-0113-7

**Published:** 2018-07-02

**Authors:** Satya V. V. N. Kothapalli, Ari Partanen, Lifei Zhu, Michael B. Altman, H. Michael Gach, Dennis E. Hallahan, Hong Chen

**Affiliations:** 1 Department of Biomedical Engineering, Washington University in St. Louis, St. Louis, MO USA; 2 Profound Medical Inc, Mississauga, Canada; 3 Department of Radiation Oncology, Washington University School of Medicine, St. Louis, MO USA; 4 Mallinckrodt Institute of Radiology, Washington University School of Medicine, St. Louis, MO USA

**Keywords:** MR-guided high-intensity focused ultrasound, MR-HIFU, Phased array transducer, Acoustic field mapping, Acoustic characterization, Fiber-optic hydrophone

## Abstract

**Background:**

With the expanding applications of magnetic resonance-guided high-intensity focused ultrasound (MR-HIFU), there is an urgent need for a convenient, reliable, and fast acoustic pressure field measurement method to aid treatment protocol design, ensure consistent and safe operation of the transducer, and facilitate regulatory approval of new techniques. Herein, we report a method for acoustic pressure field characterization of MR-HIFU systems with multi-element phased array transducers. This method integrates fiber-optic hydrophone measurements and electronic steering of the ultrasound beam with MRI-assisted HIFU focus alignment to the fiber tip.

**Methods:**

A clinical MR-HIFU system (Sonalleve V2, Profound Medical Inc., Mississauga, Canada) was used to assess the proposed method. A fiber-optic hydrophone was submerged in a degassed water bath, and the fiber tip location was traced using MRI. Subsequently, the nominal transducer focal point indicated on the MR-HIFU therapy planning software was positioned at the fiber tip, and the HIFU focus was electronically steered around the fiber tip within a 3D volume for 3D pressure field mapping, eliminating the need for an additional, expensive, and MRI-compatible 3D positioning stage. The peak positive and negative pressures were measured at the focus and validated using a standard hydrophone measurement setup outside the MRI magnet room.

**Results:**

We found that the initial MRI-assisted HIFU focus alignment had an average offset of 2.23 ± 1.33 mm from the fiber tip as identified by the 3D pressure field mapping. MRI guidance and electronic beam steering allowed 3D focus localization within ~ 1 h, i.e., faster than the typical time required using the standard laboratory setup (~ 3–4 h). Acoustic pressures measured using the proposed method were not significantly different from those obtained with the standard laboratory hydrophone measurements.

**Conclusions:**

In conclusion, our method offers a convenient, reliable, and fast acoustic pressure field characterization tool for MR-HIFU systems with phased array transducers.

## Background

Magnetic resonance-guided high-intensity focused ultrasound (MR-HIFU) integrates noninvasive and localized HIFU therapy with high resolution and multi-functional magnetic resonance imaging (MRI). MR-HIFU provides a technology platform to develop a wide range of new therapies [1]. With expanding applications of MR-HIFU, there is a need for HIFU acoustic pressure characterization tools to aid treatment protocol design, ensure consistent and safe device operation, and facilitate regulatory approval of new techniques [2]. While various methods have been proposed for MR-HIFU acoustic characterization, hydrophone measurements are considered the gold standard for direct acoustic pressure measurements [3]. Hydrophone measurements of acoustic fields have a long history, predating the introduction of HIFU. Various ultrasound hydrophone measurement devices were introduced as early as 1970s and 1980s, such as small piezoelectric hydrophone probes [4], polyvinylidene fluoride needle hydrophones [5], polymer membrane hydrophones [6], and fiber-optic hydrophone [7]. A needle hydrophone or membrane hydrophone is normally used for lower pressure measurements, while more robust sensors, such as a fiber-optic hydrophone [8], are used for high-pressure measurements. The standard laboratory hydrophone setup requires either the hydrophone or the ultrasound transducer to be translated in coupling liquid medium relative to each other using a precise 3D positioning system. Particularly, hydrophone-based characterization of a clinical phased array HIFU transducer is associated with two technical challenges: standard laboratory hydrophone equipment is not MRI-compatible, and the bore of a clinical MRI scanner has limited space to fit those pieces of equipment. Owing to these challenges, in previous studies the HIFU patient table was moved outside the magnet fringe field, and a heavy, high-precision, 3D positioning stage was placed on the table for pressure measurements [9–11]. That procedure is complicated and requires a 3D positioning stage, which is neither feasible nor economical for most clinical MR-HIFU facilities. ter Haar et al. [12] proposed an MRI-compatible positioning system for hydrophone measurements inside the magnet room. However, designing a high-precision MRI-compatible positioning system is technically challenging and expensive.

Previously, we demonstrated using the integrated robotic positioner within the HIFU patient table for acoustic characterization of a clinical MR-HIFU system inside the MR bore [13]. In that method, the transducer was translated against a fiber-optic hydrophone fixed within a water tank, and pressures at different transducer positions were acquired. Measurements obtained using the prior method were compared to hydrophone measurements performed outside the magnet. However, this method has two drawbacks: (1) each acquisition took a relatively long time (7.6 s) for initialization, transducer positioning, sonication, and hydrophone measurement, coupled with a wait time between consecutive sonication locations to prepare for the exposure and measurement at the next location, and (2) the robotic positioner in the HIFU table provides limited spatial resolution compared to the high-precision positioning stage used in the laboratory hydrophone setup.

The objective of this study was to develop and characterize a convenient, reliable, and fast method for hydrophone-based acoustic pressure measurements inside the MRI bore using a clinical MR-HIFU system with a multi-element phased array transducer. The proposed method is based on 3D electronic beam steering of the HIFU focus to accelerate the measurements and improve the spatial resolution of the measurements without the need for additional, expensive, and MRI-compatible hardware. This method is characterized by the: (1) Use of a fiber-optic hydrophone for performing measurements inside the MRI bore; (2) MRI-assisted alignment of the HIFU focus at the fiber tip using the MR-HIFU therapy planning software; and (3) Utilization of electronic beam steering to scan the focus relative to the fixed hydrophone tip location for 3D acoustic pressure field mapping. The measurements obtained with the proposed method were validated using standard laboratory hydrophone measurements conducted outside the MRI magnet room.

## Methods

### Experiment setup

**Fig. 1:**
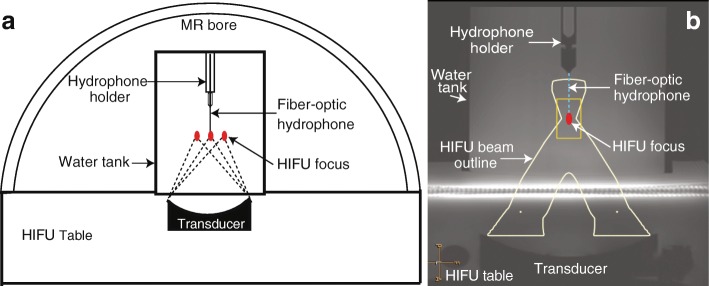
**a** Schematic representation of the experiment setup. The focal point location of the HIFU transducer was controlled using electronic steering, i.e., by adjusting the phases of the driving signals for individual elements. The transducer could also be translated using MRI-compatible motors and encoders integrated into the HIFU patient table. The patient table above the HIFU transducer was sealed with a 50 μm thick Mylar membrane to provide an acoustic window. A cylindrical, acrylic water tank (diameter 15 cm, height 30 cm), sealed on the bottom with a polyester film (McMaster-Carr, Atlanta, GA, USA), was placed on top of the acoustic window and filled with degassed, distilled water. The hydrophone was fixed within the water tank, while the HIFU focus was electronically steered around the fiber tip for 3D pressure field mapping. **b** T_2_-weighted MRI of the experimental setup. The hydrophone holder location was traced on MRI, and the fiber location (dashed line) was projected based on the distance from the hydrophone tip to the holder as measured before submerging the assembly into the water tank. The nominal HIFU focus was positioned at the fiber tip using the HIFU therapy planning software, as illustrated by the drawing of the HIFU beam outline (the beam outline is shown for illustrative purposes only and may not accurately represent the actual pressure field distribution) and the nominal HIFU focus

A detailed description of the experimental setup can be found in a prior publication [13]. A clinical MR-HIFU system (Sonalleve V2, Profound Medical Inc., Mississauga, Canada) was used in this study. The Sonalleve MR-HIFU system included a transportable patient table with an embedded-ultrasound transducer and robotic positioner, a generator cabinet, and a dedicated workstation with therapy planning software. The phased-array transducer consisted of 256 individually controllable elements with the diameter of each element being 6.6 mm. The transducer had a 135.9-mm aperture and a 140-mm radius of curvature. A fiber-optic hydrophone (HFO690, Onda Corp, Sunnyvale, CA, USA) was fixed inside a water tank using an in-house built acrylic holder (Fig. 1). The hydrophone setup included a ~ 20 m long optical fiber, allowing the MRI-incompatible hydrophone control unit to be located outside the magnet room. A digitizer card (Dynamic Signals LLC, Lockport, IL, USA) operating at a sampling frequency of 100 MHz was used to acquire hydrophone voltage data.

### Hydrophone measurements inside the MRI suite

The HIFU patient table together with the hydrophone setup was docked into the MRI bore (Ingenia 1.5 T, Philips, Best, the Netherlands). Standard MRI sequences were then used to localize the fiber tip and verify acoustic coupling between the acoustic window and the water tank.

The water tank setup and hydrophone assembly were surveyed using fast T_1_-weighted MRI to aid plan subsequent high-resolution MRI. A bubble detection MRI sequence was performed to verify that no gas bubbles were trapped between the water tank and the acoustic window. T_2_-weighted MRI was performed to guide the focus alignment at the tip of the fiber-optic hydrophone. The fiber tip (diameter 100 μm) was an order of magnitude smaller than the MRI voxel size, and thus invisible on MRI. However, the hydrophone holder was clearly visible (Fig. 1b), allowing fiber tip location projection by measuring the distance from the holder to the fiber tip before submerging the hydrophone assembly inside the water tank. The MR images were transferred to the therapy planning console for subsequent HIFU transducer positioning and nominal focus placement at the fiber tip using the measured distance from the hydrophone holder to the fiber tip (Fig. 1b).

For 3D acoustic pressure field mapping, the HIFU focus was electronically steered relative to the fixed fiber tip location. First, a relative coarse scan within a larger volume (5 × 5 × 20 mm^3^) was performed in a step size of 2 mm along the beam path (Z-axis) and 0.5 mm across the beam path (X- and Y-axes). This procedure was repeated three times to assess MRI-assisted focus alignment relative to the fiber. The time needed to perform measurements at each location was 2.8 s for initialization, sonication, hydrophone measurement, and a wait time for exposure and measurement preparation at the next location. After fiber tip localization based on the coarse scan, the transducer was repositioned with its focus aligned at the identified fiber tip location. Then, a finer scan was performed within a smaller volume (1.5 × 1.5 × 15 mm^3^) with step sizes of 1.5 mm and 0.15 mm along and across the beam path, respectively. After precise fiber tip localization through the finer scan, acoustic pressure waveforms at the focus were acquired for nominal acoustic powers ranging from 50 W to 500 W at 50 W intervals. Throughout the experiments, the HIFU transducer was operated at 1.2 MHz with a pulse repetition frequency of 12 Hz and a pulse length of 40 cycles. A custom MATLAB program (version 7.14, The Mathworks Inc., Natick, MA, USA) integrating MatHIFU [14], a MATLAB toolbox for real-time control of the Sonalleve MR-HIFU system, was used to control the HIFU beam steering, transducer positioning, HIFU pulse parameters, and hydrophone signal acquisition.

### Standard laboratory hydrophone measurements

A standard laboratory setup was used to perform hydrophone measurements outside the magnet room to validate measurements obtained inside the MRI bore. In these experiments, the HIFU table was located outside the magnet room, and a high precision Bislide^®^ 3D positioning unit (Velmex, Bloomfield, NY) with stepper motors to control hydrophone movements was placed above the HIFU table. A custom Matlab program (version 7.14, The Mathworks Inc., Natick, MA, USA) was used to control the motors of the positioning unit and to acquire hydrophone data during HIFU exposures. The focal distance was estimated to be 60 mm above the water tank bottom when the transducer was at its home position (transducer origin position at the center of the acoustic window). The 60-mm distance was calculated based on the known focal length of the transducer (140 mm) and the known distance from the transducer to the acoustic window (80 mm). To speed up focus localization, the tip of the fiber optic hydrophone was placed approximately 60 mm above the bottom of the water tank before performing measurements. Multiple iterations of 2D scans were performed in XY and YZ planes to identify HIFU focus location, starting with scanning an area of 40×40 mm^2^ in a step size of 1 mm, followed by gradually decreasing the area with decreasing step sizes, and ending with an area of 2 × 2 mm^2^ in a step size of 0.2 mm for XY plane and 2 × 15 mm^2^ in a step size of 0.2 mm for YZ plane. After focus localization, pressures were measured at the focus in triplicate at nominal acoustic powers between 50 and 500 W at 50 W intervals. Moreover, acoustic pressures as a function of electronic steering distance along each axis were measured by electronically steering the focus along the beam path (Z-axis) within a range of 35 mm and across the beam path (X-axis) within a range of 25 mm. At each steering distance, the hydrophone was moved by the same distance to be aligned with the focus, and a 2D field scan (area 1 × 1 mm^2^, step size 0.05 mm) was performed at an acoustic power of 100 W to find the exact focus location and measure the peak positive and negative pressures.

### Data processing

The hydrophone voltage data were converted to acoustic pressures using the manufacturer-provided calibration program based on the Fresnel formula [15]. Peak positive (*p*^+^) and peak negative pressures (*p*^−^) based on individual measurements were calculated from the mean of the maximum positive and negative pressures across the 10th to 30th cycles to avoid the ramp-up and ramp-down parts of the pressure waveform (Kothapalli et al. [13]). The resulting peak positive and negative pressure maps were linearly interpolated with a factor of 10 and plotted in 2D or 3D.

### Statistical analysis

Statistical analysis was performed using GraphPad Prism (Version 6.04, La Jolla, CA, USA). Group variation was described as mean ± standard deviation. Differences in acoustic pressure measurements between the proposed method and the standard method were analyzed using two-way ANOVA followed by Sidak multiple comparison tests. A *P*-value < 0.05 was considered to represent a significant difference.

## Results

**Fig. 2:**
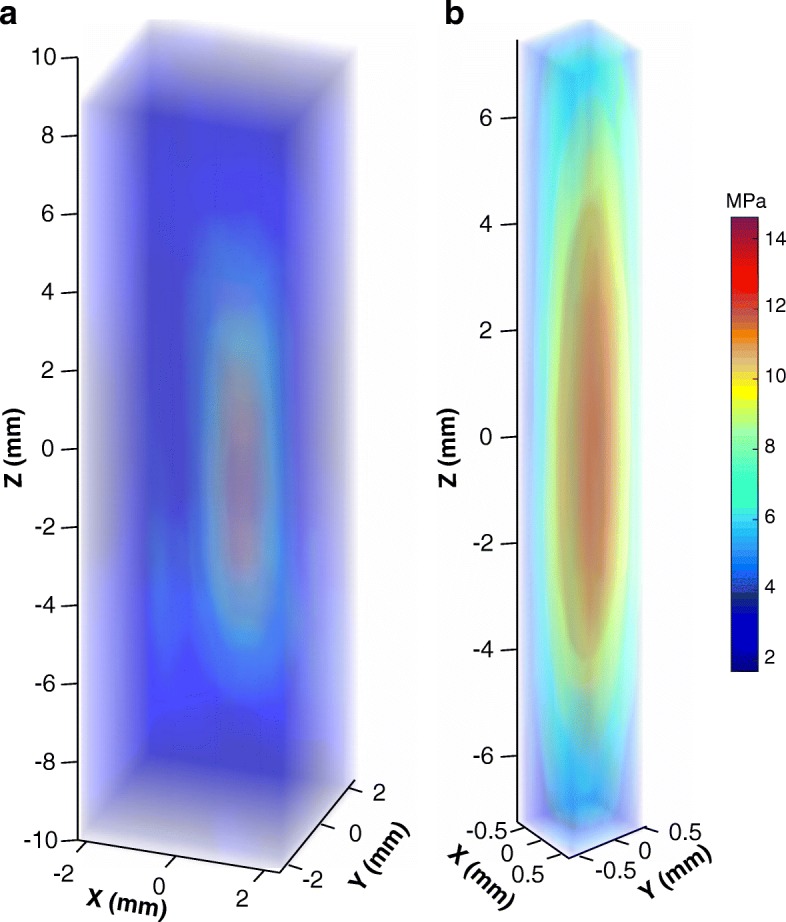
**a** A representative example of a 3D positive acoustic pressure field map within a 5 × 5 × 20 mm^3^ volume. The average offsets between the projected and identified fiber tip location over three repeated measurements were 0.7 ± 1.2 mm, 2.0 ± 0.5 mm, and 0.7 ± 0.3 mm along the X, Y, and Z axes, respectively, resulting in an average 3D offset of 2.23 ± 1.33 mm. **b** A representative example of a 3D positive acoustic pressure field map within a 1.5 × 1.5 × 15 mm^3^ volume after the transducer was repositioned to correct for the offset. Both measurements were acquired inside the MRI bore and performed at an acoustic power of 100 W, and the pressure maps were linearly interpolated with an interpolation factor of 10

Figure 2a shows a representative, volumetric acoustic pressure field map acquired inside the MRI bore after placing the nominal HIFU focus at the projected fiber tip location using the MR-HIFU therapy planning software. This plot indicates a spatial offset between the projected and identified fiber tip location. The average 3D offset was 2.23 ± 1.33 mm. After coarse fiber tip localization, the transducer was repositioned to correct for the offset, and a finer scan was performed (Fig. 2b). MRI guidance together with electronic beam steering allowed precise fiber tip localization and HIFU focus alignment within 1 h without the need for any additional positioning hardware. In comparison, focus localization took approximately 3–4 h using the standard method.

**Fig. 3:**
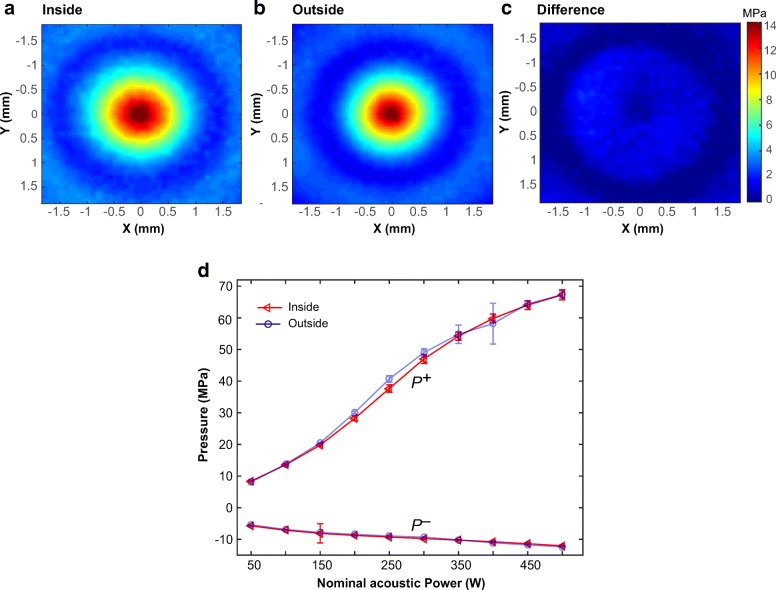
Representative examples of 2D positive pressure maps across the focus in XY plane, measured **a** inside the magnet bore and **b** outside the magnet room at a nominal acoustic power of 100 W. **c** The absolute pressure difference map between inside and outside measurements. **d** The peak positive pressures (*p*^+^, top two curves) and peak negative pressures (*p*^−^, bottom two curves) obtained inside the magnet bore and outside the magnet room at nominal acoustic powers of 50–500 W. Triangles and circles represent pressures acquired outside the magnet room and inside the magnet bore, respectively

Figure 3 demonstrates a pressure measurement comparison between the proposed method and the standard method at a nominal acoustic power of 100 W. Figure 3a and b display 2D peak positive pressure maps in the XY plane (perpendicular to beam path) obtained using the proposed and standard methods at 100 W, respectively. Figure 3c illustrates the pressure difference, i.e., the absolute pressure values measured inside the magnet bore (Fig. 3a) subtracted by pressure values measured outside the magnet room (Fig. 3b). While no statistically significant difference was found, the average difference was 0.71 ± 0.63 MPa. Figure 3d illustrates the mean and standard deviation of *p*^+^ and *p*^−^ obtained using the two methods at various acoustic power settings.

**Fig. 4:**
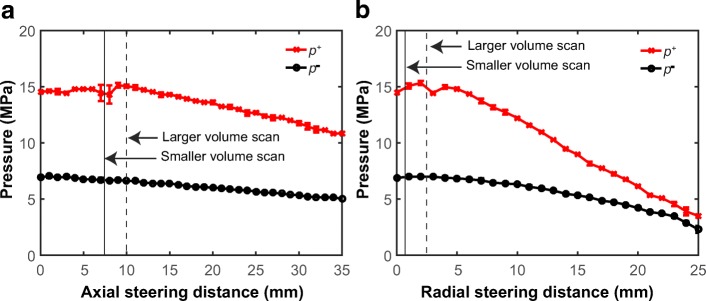
The peak positive (*p*^+^) and peak negative (*p*^−^) pressures as a function of electronic beam steering distance along **a** axial direction (Z-axis) and **b** radial direction (X-axis). The maximum steering distances used to perform the larger volume and smaller volume scans are indicated with dashed and solid vertical lines, respectively. These measurements were obtained outside the magnet room using the standard hydrophone measurement method at a nominal acoustic power of 100 W

Figure 4 presents the peak positive (*p*^+^) and peak negative (|*p*^−^|) pressures measured at different beam steering distances along axial (Z) and radial (X) direction outside the magnet. The maximum beam steering distance (the distance from the furthest measurement point to the origin) used in the larger volume pressure field mapping (Fig. 2a) was 10 mm in the axial direction and 2.5 mm in the radial direction (indicated as dashed lines in Fig. 4). The maximum beam steering distance used in the smaller volume pressure field mapping (Fig. 2b) was 7.5 mm in the axial direction and 0.75 mm in the radial direction (indicated as solid lines in Fig. 4). The *p*^+^ measured at all these four steering distances were within the range of 2.6–4.5% of the corresponding *p*^+^ measured without beam steering. The *p*^−^ measured at these four steering distances were within the range of 1.6–4.7% of the corresponding *p*^−^ measured without beam steering.

## Discussion

We presented and evaluated a method for acoustic pressure field characterization of MR-HIFU systems with multi-element phased array transducers. This method used a fiber-optic hydrophone in combination with MRI-assisted fiber tip localization and electronic beam steering. The proposed method allows convenient, reliable, and fast acoustic pressure field characterization of MR-HIFU systems with multi-element transducer arrays inside the MRI bore, without the need for additional positioning hardware.

Characterizing the HIFU acoustic pressure field is important to ensure safe and consistent operation of the system. The standard pieces of hydrophone equipment are bulky and not designed to be MR compatible, thus preventing acoustic characterization of an MR-HIFU system inside the magnet bore. Therefore, all previously reported hydrophone measurements of MR-HIFU systems were performed outside the magnet fringe field [9–11]. However, the complicated procedure involved in this process has prevented many clinical MR-HIFU sites from performing the measurements. To address those issues, our previous work explored the feasibility of using the robotic positioner integrated into the HIFU table instead of an external positioning system for acoustic characterization of a clinical MR-HIFU system inside the MR bore [13]. The integrated positioner consists of five motors, allowing 3D transducer translation to scan the HIFU focus against a fixed fiber-optic hydrophone. In contrast, the method proposed in this study uses electronic beam steering to scan the HIFU focus against the fiber-optic hydrophone. This new method provides a more rapid fiber tip localization and acoustic field characterization while eliminating the need for an additional 3D positioning system by using electronic beam steering, an inherent capability of a multi-element phased array transducer.

Electronic beam steering has two unique advantages over mechanical translation for acoustic field characterization: (1) high spatial resolution (minimal distance between two adjacent points) and (2) high temporal resolution (minimal time between two sequential measurements). The spatial resolution is limited by the resolution of the motors that drive the 3D stage. Thus, expensive high precision motors are required in standard laboratory hydrophone measurement systems. Previously, we used the motors integrated within the HIFU table to mechanically position the transducer for hydrophone measurements inside the MR bore (Kothapalli et al. [13]). However, the built-in motors have limited positioning precision. The new method proposed herein uses electronic beam steering to translate the HIFU focus in 3D, overcoming the spatial precision limitations posed by mechanical motors. The temporal resolution of the standard laboratory hydrophone measurements is limited by the vibration of the hydrophone during scanning and water perturbations generated by hydrophone movement in the water tank. A wait time of 1 s was applied in our study to allow the hydrophone to stabilize and water to settle before measuring at a new location. It is worth to point out that this wait time can be potentially shortened by successive waveform captures and setting a threshold for acceptable waveform variability, e.g., the measured pressures of three successive waveforms must vary by less than 3%. Nevertheless, measurements using the built-in motors took 7.2 s for each acquisition, which was the time needed for initialization, transducer positioning, sonication, and hydrophone measurement, coupled with a wait time between consecutive sonication locations for exposure and measurement preparation at the next location. For electronic beam steering, this time was shortened to 2.8 s for initialization, sonication, hydrophone measurement, and wait time. When only considering time for initial focus localization, MRI guidance with electronic beam steering allowed focus localization within ~ 1 h, while the time needed to identify the focus with the standard laboratory setup without imaging guidance was 3–4 h. In addition, extra time is required to move the HIFU patient table outside the MR suite (moving the HIFU patient table outside the MR suite may not even be a feasible option for some clinical sites) and to position the 3D stage onto the table when using the standard laboratory setup. The pressure measurements using the beam steering method were validated with measurements obtained using standard laboratory hydrophone setup.

The main limitation of the electronic beam steering-based hydrophone measurement method is that all phased array transducers have limited steering range, above which a pressure reduction occurs at the focus (Fig. 4). We overcame this limitation by integrating electronic beam steering with the MRI-assisted alignment of the nominal transducer focal point at the fiber tip using the MR-HIFU therapy planning software, which allowed us to localize the fiber tip with the maximum steering distances of 2.5 mm in the radial direction and 10 mm in the axial direction. Within such relatively small steering distance, the pressure reduction was less than 4.7%, which is within the measurement uncertainty of typical hydrophone measurement [16, 17]. When large steering ranges are required, the proposed method can still be used when a calibration of the pressure reduction similar as shown in Fig. 4 is performed. From the calibration curve, we can calculate the percentage of pressure reduction at each steering distance. Each pressure measured using the beam steering method can then be compensated using the corresponding percentage of pressure reductions to estimate the actual pressure at each spatial location of the acoustic field.

## Conclusions

Our proposed method that integrates fiber-optic hydrophone measurements with MRI-assisted fiber tip localization and electronic beam-steering provides a convenient, reliable, fast, and economical MR-HIFU acoustic pressure field characterization inside the magnet bore. This method can be further developed as a robust quality assurance tool to perform routine acoustic characterization of MR-HIFU systems incorporating multi-element phased array transducers and can be combined with computational models to estimate in situ acoustic pressures in tissue.

## Abbreviations

HIFU: High-intensity focused ultrasound
MR-HIFU: Magnetic resonance-guided high-intensity focused ultrasound
MRI: Magnetic resonance imaging
